# Gatekeeping and referral of patients holding private health insurance: a survey among general practitioners in Norway

**DOI:** 10.1080/02813432.2024.2380923

**Published:** 2024-07-22

**Authors:** Jørgen Breivold, Karin Isaksson Rø, Stein Nilsen, Merethe Kristine Kousgaard Andersen, Jørgen Nexøe, Stefán Hjörleifsson

**Affiliations:** a Department of Global Public Health and Primary Care, University of Bergen, Norway; b Institute for Studies of the Medical Profession, The Norwegian Medical Association, Oslo, Norway; c Research Unit for General Practice, Norwegian Labour and Welfare Administration, Norway; d Department of Public Health, Research Unit of General Practice, University of Southern Denmark, Odense, Denmark; e Ullerslev Medical Center, Ullerslev, Denmark

**Keywords:** General practice, gatekeeping, decision making, referrals, health insurance, voluntary, survey, medical overuse

## Abstract

**Objective:**

Private health insurance is becoming more common in Norway. The aim of this study was to investigate GPs’ opinions on private health insurance, and their experiences from consultations where health insurance can affect decisions about referring.

**Design:**

A web based cross-sectional survey.

**Setting:**

Norwegian general practice.

**Subjects:**

All GPs in Norway were in 2019 invited to participate in an online survey.

**Main outcome measures:**

The GPs’ opinions and experiences regarding health insurance were reported as proportions. Multiple logistic regression was used to test associations between how frequently GPs refer patients without further considerations and variables concerning their characteristics, opinions, and experiences.

**Results:**

Of 1,309 GPs (response rate 27%), 93% stated that private health insurance raises the risk of overtreatment and 90% considered such insurance to contribute to inequality in health. Frequently being pressured to refer in the absence of a medical indication was reported by 42%. Moreover, 28% often or always chose to refer patients without further consideration, and this was associated with perceptions of pressure with an adjusted odds ratio (AOR) of 3.80, 95% confidence interval (CI) 2.73–5.29, and unpleasant reactions from patients following refusals (AOR 1.63, 95% CI 1.14–2.33).

**Conclusion:**

Although most participating GPs associated private health insurance with overtreatment and inequality in health, more than one in four choose to refer without further consideration. GPs’ experience of pressure to refer and negative reactions from patients when they consider referrals not to be medically indicated, raises the risk of medical overuse for patients holding private health insurance.

## KEY POINTS

- Although most GPs had negative opinions regarding private health insurance, more than one quarter frequently referred insurance holders without further considerations.
- Perceived pressure and negative reactions from patients were associated with accommodating requests rather than acting as a gatekeeper.
- Private health insurance challenges the gatekeeping role of GPs in Norway and raises the risk of medical overuse.

## Introduction

Universal health care systems with strong primary care are associated with better public health and recommended as a global standard by the World Health Organization [1]. In Norway, all residents are covered by a National Insurance Scheme financed through taxation. This extensive welfare system provides universal health care and social services with relatively low co-payments [2]. In international comparisons, the Norwegian health care system stands out as one of the best [3]. Access to specialist care is regulated through gatekeeping by general practitioners (GPs) [2]. Gatekeeping is universally associated with efficient use of medical resources, lower health care expenses and protection against adverse effects of medical overuse [4,5].

Different private for-profit healthcare facilities exist alongside public health care in Norway [2], and the private sector has seen a significant growth in the last two decades [6]. Appointments with private specialists are expensive, but a large part of activities in private health care is financed by private health insurance. The growth of private services can thus mainly be explained by the rapidly increasing number of residents in Norway holding a private health insurance, mostly paid for by their employers. In 2020, 12% of the whole population or 25% of the workforce were covered by such insurance [6]. Private health insurance mainly covers the same medical services as those available in the public system, albeit with much shorter waiting times [7]. Most private health insurance companies in Norway require a referral from a GP for specialist appointments [8].

In a Danish study from 2017, 46% of the participating GPs reported feeling pressure to refer patients holding private health insurance despite absent or weak medical indication, and that they sometimes referred these patients without further examination or questioning [9]. This occurred although most of the GPs associated private health insurance with overtreatment and inequality in health. The Danish health care system is publicly financed, and GPs hold a similar gatekeeping role as in Norway [2].

The demand for health care services is expected to continue to rise in Norway [10]. In the face of this challenge, GPs may play an important role as gatekeepers, prioritizing healthcare resources and protecting patients against the harms that medical overuse may cause [11]. Therefore, more knowledge about how private health insurance challenges the gatekeeping role of GPs is needed. The aim of this study was to investigate Norwegian GPs’ opinions on private health insurance in general, and their experiences from consultations where such insurance can affect decisions about referring.

## Methods

### Design

A cross-sectional survey was developed by the authors JB, SH, and MA, based on a questionnaire used by MA, JN and co-authors in Denmark in 2011 [9]. A pilot version of the questionnaire was tested among eight GPs recruited from the first author’s professional network. Based on their comments, some of the questions were adjusted for increased clarity.

### Setting

In December 2019, the Norwegian College of General Practice distributed an electronic questionnaire to all its members about referrals related to private health insurance. A reminder was sent after one month. Participants working as one of the 4884 GPs among the 7504 college members were identified by a question in the questionnaire.

### Variables

The respondents answered a comprehensive questionnaire. In this paper, we focus on topics concerning GPs’ opinion of private health insurance, and experiences with consultations where the patients’ private health insurance could influence decisions regarding referrals.

Self-reported GP characteristics used in the logistic regression analyses were sex, time after completing medical undergraduate education, GP-specialist status, employment status, length of patient list, and urbanity of office location. We also asked whether the GPs held private health insurance themselves and the average number of consultations per month where the main reason for the encounter was a request from the patient for referral related to a private health insurance.

To investigate a situation where GPs choose not to act as a gatekeeper, we asked how often they would refer patients with private health insurance who requested a referral, without doing further examination or questioning. This was used as an outcome variable in the logistic regression analyses and dichotomized as 1 = always/often and 0 = never/seldom/sometimes. For this variable and other variables where we have used five-point Likert scales, the middle or neutral response option was dichotomized to the baseline group (=0). This approach ensured that the target group (=1) clearly comprised only those attitudes and experiences we wanted to investigate for statistical associations.

The participants’ opinions regarding the effects of private health insurance on inequity in health, overtreatment, benefit for society and prioritized healthcare access were also investigated. In the analysis we dichotomized the responses as 1= totally/partly agree and 0 = totally/partly disagree/neither agree nor disagree. We also asked the GPs if they would prefer that these referrals were handled by doctors employed by the insurance companies.

With five experience variables, we explored the frequency of (i) taking private health insurance into consideration when referring, (ii) claiming a separate charge, (iii) feeling pressured to refer patients with private health insurance for treatment or investigations in the absence of medical indication, (iv) experiencing patients holding private insurance as insisting and (v) being met with negative reactions from patients after refusals based on professional reasons. Those variables were dichotomized in the same way as the outcome variable.

To examine whether missing data should be imputed we conducted a missing completely at random (MCAR)-test as well as comparing the presented data with an alternative model with complete case analysis due to listwise deletion.

### Statistical analyses

Descriptive statistics with means and proportions were used to describe the sample. All variables were checked for multicollinearity calculating Spearman’s rank correlation. Preliminary bivariate logistic regressions were conducted to analyze the relationship between single predictors and referring patients without further considerations (*p* < 0.05). Selection of predictors into the final adjusted multiple logistic regression model was based on a stepwise forward hierarchical manual method, starting with a model containing only the covariate variables, sex, time after completing medical undergraduate education, list size and urbanity of office location. Variables were entered in a hierarchical inclusion order, until the p-value for the regression model was > 0.05 [12,13]. We calculated odds ratios and confidence intervals (CI) and considered *p*-values of <0.05 statistically significant. We used STATA ™ statistical software version 17.0 for the regression analysis.

## Results

### Descriptive statistics

#### Characteristics of the respondents

The survey was answered by 1484 members of the Norwegian College of General Practice. Non-GPs and retired GPs were excluded. As official statistics regarding the GP-population in Norway were available only for GPs who graduated after 1975, we also excluded respondents wo did not meet this criterion to ensure an accurate measure of representativeness. A total of 1,309 respondents were included in the analysis, comprising 27% of all practicing GPs in Norway. 53% of respondents were male and the mean time after graduation was 19.5 years. The characteristics of participating GPs compared with all Norwegian GPs are presented in Table 1.

**Table 1.** Baseline characteristics of the respondents and the population of GPs in Norway in 2019.

	Respondents, *n* (%)	GP population*, n* (%)
Sex	1288	4858^a^
Male	685 (53%)	2700 (55%)
Female	603 (47%)	2158 (45%)
Time after completing medical undergraduate education, mean years^b^	19.5	18.3^b^
Time working in general practice, mean years	16.1	–
Certified GP specialist	1308	4884^a^
Yes	929 (71%)	3049 (62%)
No	379 (29%)	1835 (38%)
Employment status	1309	4884^a^
Private practice	1158 (88%)	4201 (86%)
Salaried/combination	151 (12%)	683 (14%)
Mean number of patients listed	1158	1084
Location of office	1307	
Urban	889 (68%)	–
Rural	418 (32%)	–
Having a private health insurance themselves	1308	
Yes	144 (11%)	–
No	1164 (89%)	–
Average number of consultations per month where the main reason for encounter was a request for referral related to a private health insurance	1306	
<1	335 (26%)	–
1–3	611 (47%)	–
4–6	253 (19%)	–
7–9	73 (5%)	–
≥10	34 3%)	–

^a^The Norwegian Directorate of Health (2019). Fastlegestatistikk [Statistics on general practitioners][internet]. Oslo: The Norwegian Directorate of Health. Available from: https://www.helsedirektoratet.no/statistikk/fastlegestatistikk.

b. Correlation coef. with age is 0.9447 (*p*-value <0.001) Reference Taraldset, A., *Statitics Norway*. 2021.

#### GPs’ opinions and experiences

**Figure 1.**
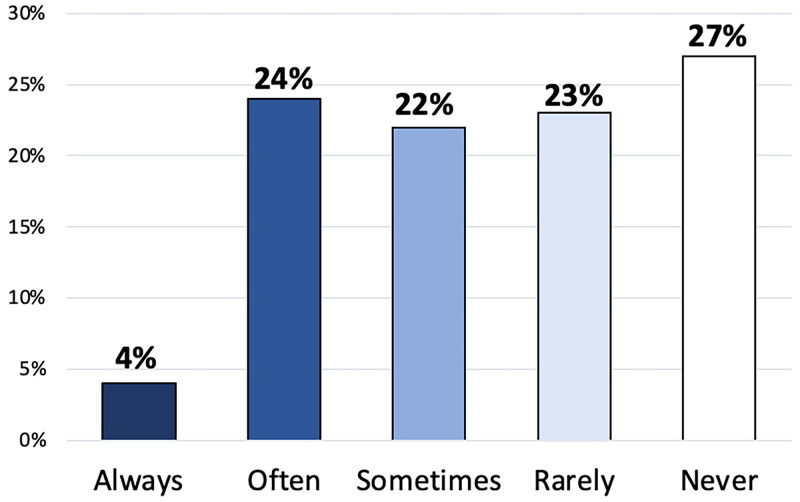
Reported frequency of GPs referring patients, who hold private health insurance and request a referral, without conducting further examination or questioning (*n* = 1308).

**Figure 2.**
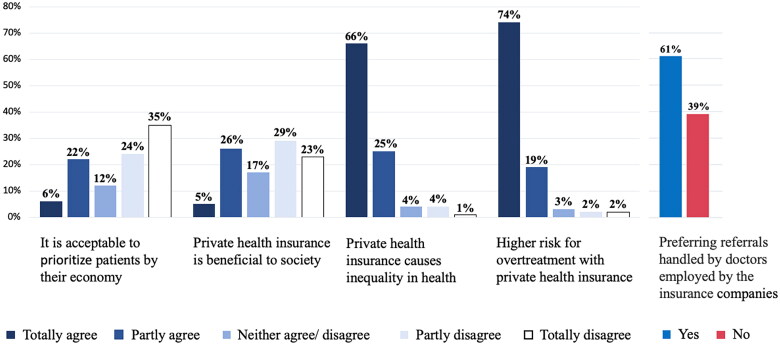
GPs’ Opinion on private health insurance (*n* = 1304).

Among the participating GPs, 28% stated that they often or always referred patients holding private health insurance without further examination or questioning, while 22% reported that they sometimes referred without such considerations (Figure 1). Most of the GPs agreed that private health insurance causes inequality in health (90%), and that patients with private health insurance risk overtreatment to a higher degree than other patients (93%) (Figure 2). One out of three (31%) agreed that private health insurance is beneficial to society and 28% agreed that it is acceptable that some patients can get treatment before others with the same needs if they can afford to pay for it or have insurance. 61% of the respondents preferred that referrals related to a private health insurance scheme were managed by doctors employed by the insurance companies.

**Figure 3.**
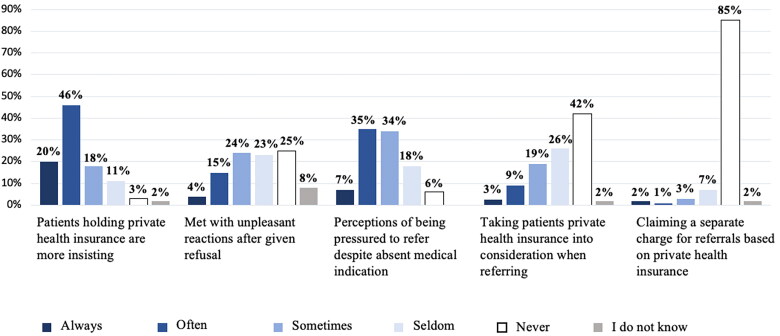
General practitioners’ experience with consultations where the patient’s private health insurance could impact on the cause of action (*n* = 1306).

42% reported that in consultations with patients holding private health insurance, they would often or always feel pressure to refer for treatment or investigations, even if they found no medical indication (Figure 3). An additional 34% reported that they sometimes experienced such pressure. 12% reported to often or always take into consideration whether or not the patient held a private health insurance when referring patients, and 66% had frequently experienced that patients who held a private health insurance were more insistent when requesting referral than patients without one. Furthermore, 18% stated that they often or always were met with unpleasant reactions such as aggression, threats, or reprisals when they on professional grounds refused to fulfill a request for referral from a patient holding private health insurance. Most of the GPs (92%) stated that they would rarely or never request a separate charge for referrals based on private health insurance.

### Logistic regression analysis

As shown in Table 2, no GP characteristics were associated with referring patients without further considerations. However, referring without further considerations correlated with finding it acceptable that some patients get treatment before others with similar health problems if they can afford to pay for it or have a private insurance with an adjusted odds ratio (AOR) of 1.55 and a 95% confidence interval (CI) 1.12–2.14. Preferring that referrals related to private health insurance were managed by doctors employed by the insurance companies themselves (AOR 1.47, 95% CI 1.06–2.03) also correlated with the outcome variable.

**Table 2.** Logistic regression analysis estimating the associations between the frequency of Norwegian GPs referring patients without further considerations if they hold a private health insurance, and GP characteristics, attitudes or experiences concerning private health insurance in 2019 (adjusted model *n* = 1126).

		Crude model				Adjusted model		
	Category (=1)	n bivariate analysis	OR	95% CI	*p-value*	OR	95% CI	*p-value*
**GP characteristics**								
Sex^a, b^	Male	1286	1.18	0.92–1.51	0.187	1.24	0.92–1.67	0.156
Time after completing medical undergraduate education^a^	Years after graduation, continuous	1307	1.00	0.99–1.01	0.576	1.02	1.01–1.03	0.002
Certified GPs^c^	Yes	1306	0.96	0.73–1.25	0.748	–		
Location of office^a, d^	Urban	1305	1.25	0.96-–1.62	0.103	1.04	0.76–1.43	0.806
Employment status^e^	Private practice	1307	1.38	0.92–2.07	0.116	–		
Number of patients listed^a, f^	>1500	1307	1.51	1.03–2.19	0.032	1.01	0.64–1.61	0.967
Have you got a private health insurance yourself^c^	Yes	1306	0.92	0.62–1.35	0.659	–		
How many consultations do you have during an average month where the patient requests a referral based on a private health insurance?^g^	≥4 consultations	1304	1.91	1.47–2.47	<0.001	–		
**Attitude variables^h^**								
Private health insurance causes inequality in health^i^	Totally/ partly agree	1303	0.99	0.66–1.50	0.970	–		
Patients with private health insurance risk overtreatment to a higher degree than patients without private health insurance^i^	Totally/ partly agree	1302	1.48	0.89–2.46	0.132	–		
In general, private health insurance is to the benefit of society because the insured person returns to work sooner^i^	Totally/ partly agree	1301	1.14	0.88–1.48	0.324	–		
It is acceptable that some patients get treatment before others with the same problem if they can afford to pay for it or have an insurance^a, i^	Totally/ partly agree	1302	1.43	1.10–1.86	0.007	1.55	1.12–2.14	0.008
Would you prefer that referrals covered by private health insurance schemes were handled by doctors employed by the insurance companies themselves?^ a, c ^	Yes	1307	1.82	1.40–2.36	<0.001	1.47	1.06–2.03	0.020
**Experience variables^h^**								
When referring patients, I take into consideration whether the patient has a private health insurance or not^a, j^	Often/ Always	1282	3.12	2.21–4.40	<0.001	2.68	1.78–4.04	<0.001
I choose to claim a separate charge when referring patients using private health insurance^j^	Often/ Always	1282	1.46	0.75–2.84	0.267	–		
As a GP I experience being under pressure to refer patients with private health insurances for examination or treatments that I consider not to be medically warranted^a, j^	Often/ Always	1306	3.96	3.06–5.11	<0.001	3.80	2.73–5.29	<0.001
Patients informing that they hold a private health insurance insist more on getting a referral than patients without one^a, j^	Often/ Always	1278	2.14	1.61–2.85	<0.001	–		
When I do not want to fulfill a request for referral from a patient holding health insurance based on professional reasons, I am met with aggression, threats, reprisals etc.^a, j^	Often/ Always	1196	2.97	2.20–3.99	<0.001	1.63	1.14–2.33	0.007

^a^) Included in the final adjusted model using stepwise forward hierarchical manual method

^b^ Responses dichotomized 1 = male, 0 = female.

^c^ Responses dichotomized 1 = yes, 0 = no.

(d) Responses dichotomized 1 = urban, 0 = rural. (e) Responses dichotomized 1 = private, 0 = salary/combined. (f) Responses dichotomized 1 = >1500 patients, 0 = <1500 patients. (g) Responses dichotomized 1 = four or more than four, 0 = less than four. (h) Possible answer ‘I do not know’ was not included in the analyses. (i) Responses dichotomized 1 = totally/partly agree, 0= totally/partly disagree/neither agree nor disagree. (j) Responses dichotomized 1 = always/often and 0 = never/infrequently/sometimes.

We found a significant association between referring patients without further considerations and perceptions of being pressured to refer patients with private health insurance for treatment or investigations without medical indication (AOR 3.80, 95% CI 2.73–5.29). Frequently being met with unpleasant reactions when refusing to refer a patient (AOR 1.63, 95% CI 1.14–2.33) and frequently taking into consideration whether the patient held private health insurance (AOR 2.68, 95% CI 1.78–4.04) was also correlated with the outcome variable of referring patients without further considerations.

## Discussion

### Principal findings

Most of the GPs who responded to this survey stated that private health insurance causes inequality in health and raises the risk of overtreatment. Nevertheless, more than one in four reported that they would often or always refer patients without further examination or questioning. This was associated with frequently perceiving pressure to refer as well as being met with unpleasant reactions after refusing to refer in the absence of a medical indication.

### Strengths and limitations

A strength of this study is that almost all GPs in Norway received an invitation to participate. The background characteristics of the respondents were similar to those of the general Norwegian GP population except that the proportion of GP specialists among the respondents was significantly higher compared with the total GP population. The response rate of 27% is low, but similar to other surveys among physicians [14]. Web-based surveys yield more complete data but have lower participation rates compared to postal surveys [15]. It was not possible to investigate reasons for not replying to the questionnaire.

The results must be interpreted in the context of the Norwegian health care system and are likely to be of most relevance in settings with a similar health care system and similar use of private health insurance. However, understanding how pressure from patients can lead to medical overuse can also be of relevance in other contexts [16].

We chose not to impute values for missing data in the analysis, being aware that this could compromise the validity of the study findings. However, a statistical model with complete case analysis gave the same results as the data presented although we did not use this model due to a lack of the MCAR-assumption [17]. To test the robustness of the dataset, we also conducted analyses using alternative regression models (forward selection and backward elimination), resulting in the same significant predictors as the stepwise hierarchical regression.

As GPs with negative experiences concerning referrals related to private health insurance may have been more likely to participate, there might be a selection bias toward a higher proportion reporting to feel pressure to refer in the absence of a medical indication or referring patients without further considerations.

### Comparison with existing literature

Our findings are generally in line with the Danish study from 2017 which inspired us to explore this topic in Norwegian general practice [9]. Most GPs in both studies believed that private health insurance causes inequality in health and raises the risk of overtreatment, and more than four in ten GPs frequently experienced being pressured to refer patients with private health insurance for treatment or investigations in the absence of a medical indication. Notably, the proportion of participants who reported to refer patients without further considerations was two and a half times higher in Norway than in Denmark [9]. This difference may be caused by any among a wide range of contextual factors, but as the study in Denmark was conducted eight years earlier than our study it is possible that GPs in Scandinavia are increasingly abdicating from the role as gatekeepers when consulted by patients with private health insurance. The Danish study concluded that insisting patients to some degree explained the contradiction between experiencing pressure to refer or referring without further considerations and believing that private health insurance leads to overtreatment and inequality in health [9]. When we included perception of pressure to refer as one of the independent variables, this correlation disappeared, indicating confounding.

The majority of the participants in our study reported either that they at least sometimes experienced pressure to refer in the absence of a medical indication *or* that they at least sometimes referred patients without further considerations. Respect for patient autonomy and shared decision making are valued in general practice settings [18]. However, patients reading customer information from the insurance companies can wrongly get the impression that they are entitled to get a referral [8]. Consumerist models of health care can challenge doctor’s obligation to act in the patient’s best interests [19]. Pressure from consumerist patients has also been found to be a driver for defensive medicine, understood as questionable and unnecessary medical activities caused by external demands [20]. GPs have better diagnostic competence than lay people [5], and low acceptance of gatekeeping in primary care may contribute to medical overuse [21].

Insurance companies in Norway have in effect made GPs responsible for gatekeeping to their services, without having negotiated an agreement with the GP Union about this. A majority of the respondents in this study stated that the insurance companies should employ their own doctors to handle this task. It is possible that even if GPs consider themselves to be gatekeepers for secondary care, they do not believe that this should include referrals to private care providers. This perspective may have contributed to the association we observed between taking into consideration whether the patient held private health insurance and referring patients without further considerations.

Discomfort related to pressure and unpleasant reactions from patients may reduce GPs’ motivation to act as gatekeepers in general, and it is known from other studies that GPs are not always comfortable with gatekeeping [22,23]. GPs sometimes choose to refer in the absence of a medical indication to avoid conflicts with their patients [22]. More than a third of GPs in a Dutch survey stated that their decisions about referring patients in general were not based on clinical indication, and two-thirds of these GPs had conceded to patients demanding a referral [24]. The authors concluded that GPs who readily satisfy patients’ demands contribute to medical overuse.

In contrast to the results from the Danish study, we found a correlation between referring patients without further considerations and accepting that patients with more economic resources can be prioritized above others [9]. Patients holding private health insurance are known to be a privileged group [25]. According to another Danish study regarding defensive medicine, GPs experienced that patients with higher socioeconomic status in general were more demanding than other patients [20]. In Europe, based on need, high-income patients are more likely to see a medical specialist, especially in countries where it is possible to buy quicker access to private specialist health care [26]. This correlation has also been demonstrated in Norway [27].

## Implications

The proportion of Norwegians who hold a private health insurance has increased further since the data for this study were collected [7]. If patients also increasingly consider themselves as entitled to services according to their own wishes, the gatekeeping function will be further challenged. This can undermine the principle of two-tiered universal health care where access to specialized care is regulated according to medical needs. It seems that society must either accept this transformation in Norwegian health care or bolster the gatekeeping role of GPs. The causes of the variation in perceived pressure and gatekeeping practice found in the current study should be explored further.

## Conclusion

Although most of the participating GPs stated that private health insurance causes inequality in health and raises the risk of overtreatment, more than one in four frequently choose to refer patients without further examination or questioning. GPs’ experiences of pressure to refer and unpleasant reactions after refusing to refer in the absence of medical indication are associated with accommodating requests rather than acting as a gatekeeper. This indicates that patients holding private health insurance are at increased risk of medical overuse.

## Ethical approval

All data from the participating GPs were anonymized. The GPs were informed in writing that participation was voluntary and that results would be used in research and published. Since the responses were anonymous, no formal medical ethics approval was required.
